# Oxyresveratrol Increases Energy Expenditure through Foxo3a-Mediated Ucp1 Induction in High-Fat-Diet-Induced Obese Mice

**DOI:** 10.3390/ijms20010026

**Published:** 2018-12-21

**Authors:** Jin Hee Choi, No-Joon Song, A Reum Lee, Dong Ho Lee, Min-Ju Seo, Suji Kim, Seo-Hyuk Chang, Dong Kwon Yang, Yu-Jin Hwang, Kyung-A Hwang, Tal Soo Ha, Ui Jeong Yun, Kye Won Park

**Affiliations:** 1 Department of Food Science and Biotechnology, Sungkyunkwan University, Suwon, Gyeonggi-do 16419, Korea; theresa159@naver.com (J.H.C.); songnj413@naver.com (N.-J.S.); areumy@icloud.com (A.R.L.); zestcoren@gmail.com (D.H.L.); smj713@naver.com (M.-J.S.); suji_11@nate.com (S.K.); adc153@naver.com (S.-H.C.); yunc@skku.edu (U.J.Y.); 2 Department of Veterinary Pharmacology and Toxicology, College of Veterinary Medicine, Chonbuk National University, Iksan, Jeollabuk-do 54596, Korea; dkyang0502@gmail.com; 3 Department of Agrofood Resources, National Institute of Agricultural Sciences, RDA, Wanju-Gun, Jeollabuk-do 55365, Korea; yjhwang1022@korea.kr (Y.-J.H.); kah366@korea.kr (K.-A.H.); 4 Department of Life Science, Deagu University, Gyeongsan, Gyeongsangbuk-do 38453, Korea; tal.ha@daegu.ac.kr

**Keywords:** oxyresveratrol, energy expenditure, brown adipocyte, Ucp1, obesity

## Abstract

The phytochemical oxyresveratrol has been shown to exert diverse biological activities including prevention of obesity. However, the exact reason underlying the anti-obese effects of oxyresveratrol is not fully understood. Here, we investigated the effects and mechanism of oxyresveratrol in adipocytes and high-fat diet (HFD)-fed obese mice. Oxyresveratrol suppressed lipid accumulation and expression of adipocyte markers during the adipocyte differentiation of 3T3-L1 and C3H10T1/2 cells. Administration of oxyresveratrol in HFD-fed obese mice prevented body-weight gains, lowered adipose tissue weights, improved lipid profiles, and increased glucose tolerance. The anti-obese effects were linked to increases in energy expenditure and higher rectal temperatures without affecting food intake, fecal lipid content, and physical activity. The increased energy expenditure by oxyresveratrol was concordant with the induction of thermogenic genes including Ucp1, and the reduction of white adipocyte selective genes in adipose tissue. Furthermore, Foxo3a was identified as an oxyresveratrol-induced gene and it mimicked the effects of oxyresveratrol for induction of thermogenic genes and suppression of white adipocyte selective genes, suggesting the role of Foxo3a in oxyresveratrol-mediated anti-obese effects. Taken together, these data show that oxyresveratrol increases energy expenditure through the induction of thermogenic genes in adipose tissue and further implicates oxyresveratrol as an ingredient and Foxo3a as a molecular target for the development of functional foods in obesity and metabolic diseases.

## 1. Introduction

Energy imbalance whereby calories consumed exceed calories expended causes obesity and abnormal excess fat accumulation [1]. The World Health Organization (WHO) estimated that 650 million adults and 340 million children aged 5–19 were obese in 2016. An increased intake of energy-dense foods and physical inactivity due to the modern lifestyle will further globally increase the prevalence of obesity in the future [2]. Obesity is also a major risk factor for diabetes, hepatic diseases, cardiovascular diseases, and certain cancers [3,4]. Although healthy diets and regular physical activity are easy choices to prevent obesity, these lifestyle changes are not easily accomplished; effective and safe therapeutic strategies are thus needed to reduce the incidence of obesity.

Increases of energy expenditure can provide interventions against obesity and its associated metabolic diseases [5,6,7]. White adipose tissue (WAT) stores energy as a form of triglycerides in a unilocular lipid droplet [8]. WAT also affects systemic metabolism by the secretion of adipokines [9]. Brown adipose tissue (BAT) can generate heat through the oxidation of fat stored in multilocular lipid droplets by high uncoupling protein1 (Ucp1) expression in mitochondria under cold exposure [7,10,11]. Beige adipocytes contain high mitochondrial contents, multilocular lipid droplets, and high levels of Ucp1 expression similar to BAT [6,7,12]. However, while brown adipocytes can be derived from progenitors that express myogenic factor (Myf5), paired box (Pax7), and engrailed (En1) in the dermomyotome, beige adipocytes are derived from precursor cells that express platelet-derived growth factor–β (PDGF-β), smooth muscle actin (SMA), and myosin heavy chain 11 (Myh11) [13]. Due to the uncoupling of respiration and adenosine triphosphate (ATP) synthesis, BAT (and beige adipocytes) increases energy expenditure and thus is considered to be a tool to prevent obesity and metabolic diseases [5,6].

Phytochemicals including berberine, butein, capsaicin, resveratrol, and curcumin can increase energy expenditure through the stimulation of thermogenic brown or beige adipocytes. Various molecular targets such as 5’ adenosine monophosphate-activated protein kinase (AMPK), Prdm4, TRPV1, and beta-adrenergic pathway are regulated by these phytochemicals for their thermogenic responses [14]. This suggests that phytochemically induced thermogenic adipocytes may be useful to combat human metabolic diseases and further present molecular targets for WAT browning and energy expenditure.

Oxyresveratrol (trans-3,5,2’,4’-tetrahydroxystilbene) found in mulberry twigs and fruits (Morus alba L.) has an additional hydroxyl group on the aromatic ring of resveratrol [15]. Oxyresveratrol displays effects in metabolism including lowering cholesterol levels, abrogating hepatic oxidative stress, antihyperlipidemic effects, and anti-obese actions [16,17,18]. However, the molecular mechanism of preventing obesity and its associated metabolic diseases is not understood. In the current study, we identified oxyresveratrol as an inducer of WAT browning in adipocytes and obese mice. Treatments of oxyresveratrol prevented obesity and improved metabolic dysregulation in high-fat diet (HFD)-fed obese mice. Oxyresveratrol increased energy expenditure without affecting food intake and physical activity. The increased energy expenditure by oxyresveratrol is manifested by sustained expression of Ucp1 and thermogenic markers in adipocytes and WATs. We further identified Foxo3a as an oxyresveratrol-regulated new thermogenic regulator in adipocytes. These results show that oxyresveratrol increases energy expenditure by acting on the stimulation of thermogenesis and further implicating its potential use in treating obesity and metabolic diseases.

## 2. Results

### 2.1. Oxyresveratrol Decreases Lipid Accumulation and Adipocyte Differentiation

Prior to testing the effects of oxyresveratrol in adipocytes, we performed a 3-(4,5-dimethylthiazol-2-yl)-2,5-diphenyltetrazolium bromide (MTT) assay in 3T3-L1 cells to select optimal doses of oxyresveratrol (Figure S1). Treatment of 3T3-L1 cells with 1–200 μM oxyresveratrol for 48 h did not significantly affect cell viability (Figure S1). Oxyresveratrol at 200 μM for 96 h exhibited cellular toxicity (Figure S1). Thus, we chose oxyresveratrol at 100 μM or lower concentrations to assess its effects on adipocytes. We differentiated 3T3-L1 cells into adipocytes in the presence of oxyresveratrol for 6 days and assessed lipid accumulation by Oil Red O staining. Oxyresveratrol dose-dependently inhibited lipid accumulation (Figure S1). The adipocyte markers of Pparγ and Fabp4 expressions were consistently suppressed by oxyresveratrol in the 3T3-L1 cells (Figure S1). Similarly, oxyresveratrol exhibited anti-lipogenic effects in the C3H10T1/2 cells (Figure S1). These data show that oxyresveratrol suppresses lipid accumulation and adipocyte differentiation in the 3T3-L1 and C3H10T1/2 cells.

### 2.2. Oxyresveratrol Prevents Weight Gain and Improves Metabolic Profiles in HFD-Fed Obese Mice

**Figure 1:**
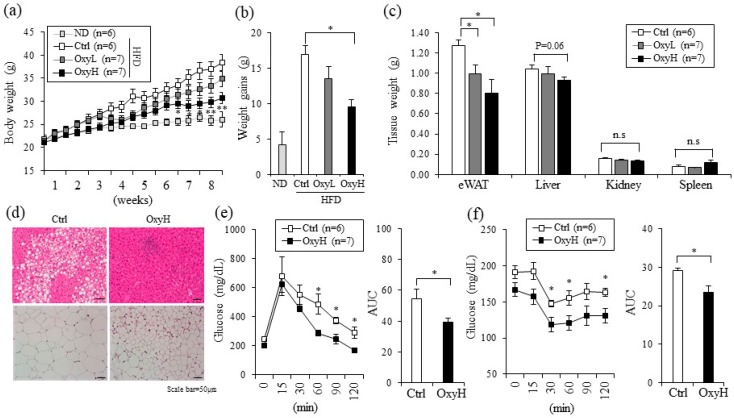
Oxyresveratrol prevents obesity and improves glucose metabolism in HFD-fed obese mice. (**a**) Body weight of vehicle control or oxyresveratrol-treated mice fed with high-fat diet (HFD) for 8 weeks. Male C57BL/6N mice were fed with a normal chow diet (ND, 20% fat) or HFD, 60% fat) and treated with vehicle control (Ctrl), oxyresveratrol (7.5 mg/kg, OxyL) or OxyH (15 mg/kg per day) for 8 weeks. (**b**) Body-weight gain of ND, HFD vehicle control (Ctrl) or oxyresveratrol-treated HFD-fed obese mice. (**c**) Epididymal fat (eWAT), liver, kidney, and spleen weights in HFD vehicle control (Ctrl) or oxyresveratrol-treated mice. (**d**) Representative hematoxylin and eosin (H&E) staining for sections of liver (top) and inguinal fat (iWAT) (bottom) from Ctrl and OxyH mice. Scale bar, 50 µm. (**e**) Glucose tolerance test (left) and area under curve (right) in Ctrl or OxyH mice. Mice were fasted for 16 h before intraperitoneal injection of glucose (2 g/kg). (**f**) Insulin tolerance test (left) and area under curve (right) in Ctrl or OxyH mice. Mice were fasted for 16 h before intraperitoneal injection insulin (0.35 U/kg). Glucose levels were measured from tail blood samples collected at the indicated time points. Data represent means ± s.e.m. Statistically significant differences between vehicle control (Ctrl) and Oxy-treated mice were determined using Student’s *t*-test (* *p* < 0.05; ** *p* < 0.005).

The in vitro anti-adipogenic effects of genes and phytochemicals are often associated with in vivo anti-obesity effects [5,19]. To test this association, we investigated the anti-obese effects of oxyresveratrol in HFD induced obese mice. Eight-week-old C57BL/6N mice were fed with an HFD (60% fat) and intraperitoneally administrated a vehicle control (Ctrl) or treated with 7.5 mg/kg/day (OxyL) or 15 mg/kg/day (OxyH) of oxyresveratrol for 8 weeks. The oxyresveratrol-treated group that was administered 7.5 mg/kg/day gained about 8% less body weight and the group that was administered 15 mg/kg/day gained 18% less body weight than the control HFD (Ctrl) group (Figure 1a,b).

Epididymal fat tissue and liver weights were significantly lower in the oxyresveratrol-treated groups than in the control groups, whereas other tissues such as spleen and kidney did not differ among these groups (Figure 1c). We also measured the serum glucose and lipid levels as these are positively associated with obesity. Plasma glucose levels decreased from 280 mg/dL in the control group to 220 in the 15 mg/kg/day oxyresveratrol (OxyH) treated groups. Serum total cholesterol and low-density lipoprotein (LDL) levels were also decreased significantly in the oxyresveratrol-treated group. Plasma fatty acid levels were significantly decreased, whereas plasma triglycerides levels tended to be lower in the oxyresveratrol (OxyH) treated mice than in the vehicle treated (Ctrl) mice (Figure S2). Hematoxylin and eosin staining revealed that large adipocytes of inguinal WAT (iWAT) and hepatic steatosis induced by HFD in the Ctrl group were blocked in the OxyH group (Figure 1d and Figure S3). Consistent with these observations, glucose tolerance and insulin tolerance were significantly improved in the OxyH mice compared to those in the Ctrl mice (Figure 1e,f). These data show that oxyresveratrol prevents weight gain and its associated metabolic complications in the HFD-fed obese mouse model.

### 2.3. Oxyresveratrol Increases Energy Expenditure

The therapeutic effects of oxyresveratrol in obesity and its associated metabolic complications can be mediated by several effects including lowering energy intake, stimulating physical activities, or increasing resting metabolic rate.

**Figure 2:**
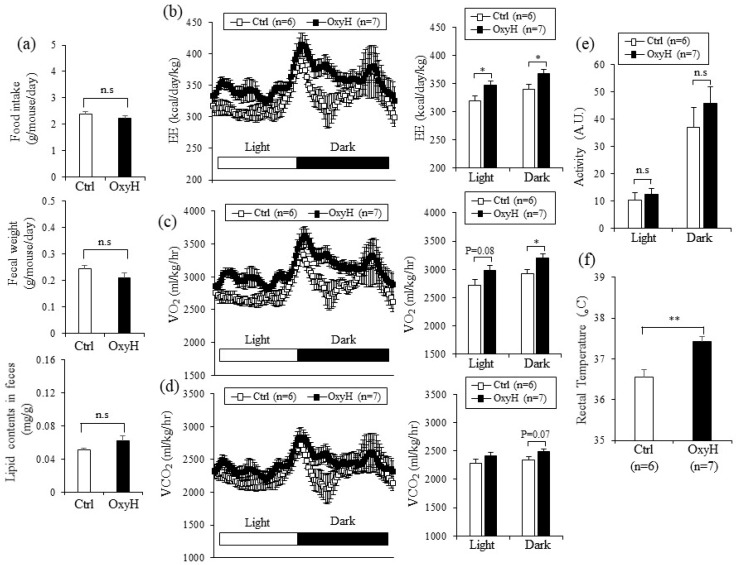
Oxyresveratrol increases energy expenditure in HFD-fed obese mice. (**a**) Male C57BL/6N mice were fed a HFD and treated with vehicle control (Ctrl) or oxyresveratrol (OxyH, 15 mg/kg per day) for 8 weeks. Food intake (top), fecal weight (middle), and lipid contents in feces (bottom) of vehicle control (Ctrl) or oxyresveratrol (OxyH)-treated obese mice. (**b**) Energy expenditure, (**c**) oxygen consumption, and (**d**) carbon dioxide production were measured in control (Ctrl, *n* = 6) or oxyresveratrol (OxyH, *n* = 7) treated mice by indirect calorimetry using Oxylet system after 3 weeks on HFD. Energy expenditure was measured before body weights started to diverge. Bar graph (right panel) represents the average energy expenditure, O_2_ consumption, and CO_2_ production in each group. (**e**) Total physical activities of control (*n* = 6) and oxyresveratrol-treated mice (*n* = 7). (**f**) Rectal temperature was measured in mice treated with control (*n* = 6) or oxyresveratrol (*n* = 7). Data represent mean ± s.e.m. and statistically significant differences between control or oxyresveratrol-treated mice were determined using Student’s *t*-test (* *p* < 0.05; ** *p* < 0.005).

To gain insight into the underlying mechanism for the anti-obese effects of oxyresveratrol, we measured food intake, physical activities, and energy expenditure. Food intake was similar among the oxyresveratrol-treated (OxyH) and control (Ctrl) mice groups (Figure 2a). Furthermore, fecal weight and fecal lipid content did not significantly differ between the mice groups, suggesting that reducing energy intake is unlikely to cause weight differences among these groups (Figure 2a). In contrast, energy expenditure (Figure 2b) and consumption of oxygen (Figure 2c) were significantly higher in the oxyresveratrol-treated mice, even before the weight differences were observed (3 weeks post-treatment). In addition, the oxyresveratrol-treated mice tended to produce more carbon dioxide (Figure 2d). However, the physical activities between these groups did not differ (Figure 2e). These data indicate that increased energy expenditure can be regarded as a primary effector for oxyresveratrol-mediated weight management in obese mice. Consistent with increased energy expenditure, rectal temperatures were higher in the oxyresveratrol-treated mice than in the control mice (Figure 2f). These results show that oxyresveratrol exerts anti-obese effects through the stimulation of energy expenditure in obese mice.

### 2.4. Oxyresveratrol Stimulates the Expression of Thermogenic Genes in Adipocytes and Adipose Tissue

**Figure 3:**
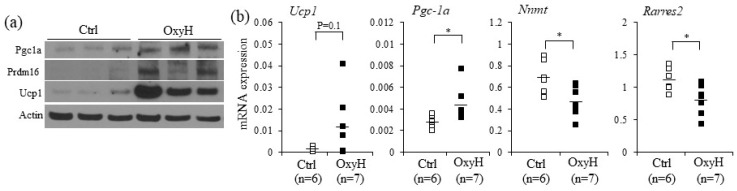
Oxyresveratrol selectively increases thermogenic adipocyte markers but decreases white adipocyte markers in white adipose tissues. Male C57BL/6N mice were fed a HFD and treated with vehicle control (Ctrl) or oxyresveratrol (OxyH, 15 mg/kg per day) for 8 weeks. (**a**) Expression of Pgc-1α, Prdm16, and Ucp1 protein expression in inguinal adipose tissue from control or oxyresveratrol-treated mice was measured by Western blotting. (**b**) Expression of white selective (*Nnmt* and *Rarres2*) and thermogenic selective genes (*Ucp1* and *Pgc-1α*) in epididymal white adipose tissue from control (*n* = 6) or oxyresveratrol-treated mice (*n* = 7) was measured by real-time PCR. Rhombus (open and closed) and bars in scatter plots represent individual mice and the average, respectively. Statistically significant differences in the control and oxyresveratrol-treated mice were determined by Student’s *t*-test (* *p* < 0.05).

Given the increased resting energy expenditure by oxyresveratrol treatment, it is plausible that oxyresveratrol may directly stimulate thermogenic gene expression in WAT. To test this possibility, we measured the expression levels of thermogenic selective and white adipose selective genes from WATs of oxyresveratrol or vehicle treated mice. Consistent with increased energy expenditure and higher rectal temperature, a major thermogenic player, the uncoupling protein1 (Ucp1) levels were higher in the oxyresveratrol-treated mice (Figure 3a and Figure S8). Similarly, thermogenic transcriptional stimulators, PR domain containing protein 16 (Prdm16) and a mitochondrial biogenesis activator Peroxisome proliferator-activated receptor gamma coactivator 1-alpha (Pgc-1α) proteins increased in the oxyresveratrol-treated mice compared to those in vehicle treated control obese mice (Figure 3a and Figure S8). Similar to the protein expression levels, we further confirmed the induction of thermogenic genes by real-time polymerase chain reaction (real-time PCR) analysis. Epididymal WAT (eWAT) in the oxyresveratrol-treated mice exhibited higher levels of Ucp1 and Pgc-1α with lower expression of white adipocyte selective genes such as Nicotinamide N-Methyltransferase (Nnmt) and Rarres2 mRNA expression (Figure 3b).

**Figure 4:**
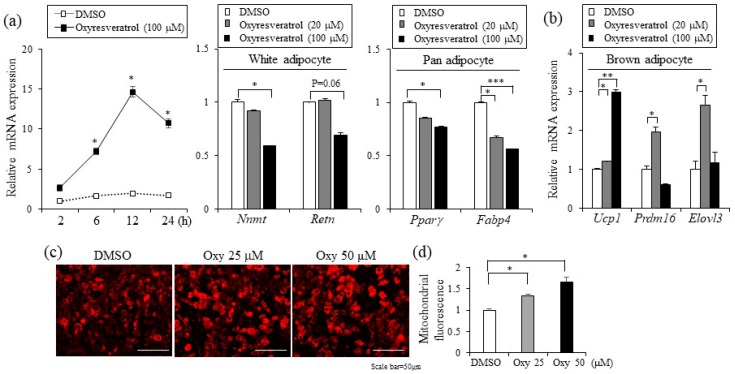
Oxyresveratrol induces Ucp1 and thermogenic genes in adipocytes. (**a**) C3H10T1/2 cells were differentiated into adipocytes and treated with oxyresveratrol for up to 24 h and the *Ucp1* expression was measured by real-time PCR. C3H10T1/2 adipocytes were treated with oxyresveratrol for 12 h, and the expression of white adipocyte selective genes (*Nnmt* and *Retn*), and general adipocyte genes (*Pparγ* and *Fabp4*) were verified by real-time PCR. (**b**) T37i brown adipocytes were treated with oxyresveratrol for 24 h, and the levels of thermogenic selective markers (*Ucp1*, *Prdm16*, and *Elovl3*) were measured. (**c**) Mitochondrial staining by mitochondria-specific CytoPainter (ab112145) in C3H10T1/2 adipocytes treated with dimethyl sulfoxide (DMSO) or oxyresveratrol for 24 h. (**d**) Quantification of mitochondrial fluorescence. Mitochondrial staining was quantified using National Institutes of Health (NIH) Image J software. Data represent means ± s.e.m. and are representative of three independent experiments. Statistical significance was determined relative to a control using the Student’s *t*-test (* *p* < 0.05; ** *p* < 0.005; *** *p* < 0.0005).

Having discovered the induction of Ucp1 and brown adipocyte selective genes by oxyresveratrol in adipose tissue, we examined cell autonomous effects of oxyresveratrol in adipocytes. To evaluate the direct impacts of oxyresveratrol on the expression levels of genes involved in thermogenic adipocytes, we treated oxyresveratrol in differentiated C3H10T1/2 adipocytes and measured the expression levels of Ucp1. Oxyresveratrol-induced Ucp1 expression with a peak at 12 h treatments (Figure 4a). Interestingly, these thermogenic genes increased at the expense of white adipose selective genes as the expression of Nicotinamide N-Methyltransferase (Nnmt) and Resistin (Retn) were suppressed. Pan-adipocyte markers Pparγ and Fabp4 were also repressed by oxyresveratrol (Figure 4a). Furthermore, the Ucp1 induction and other thermogenic adipocyte selective markers including Prdm16 and Elovl3 expression were also induced by oxyresveratrol in T37i brown adipocytes (Figure 4b). Furthermore, oxyresveratrol treatments increased mitochondrial mass in C3H10T1/2 adipocytes (Figure 4c,d). Taken together, these results strongly suggest that oxyresveratrol induces thermogenic genes in adipocytes and increases energy expenditure, leading to the prevention of obesity and metabolic dysregulation.

### 2.5. Foxo3a Induced by Oxyresveratrol Stimulates Thermogenic Gene Expression in Adipocytes

**Figure 5:**
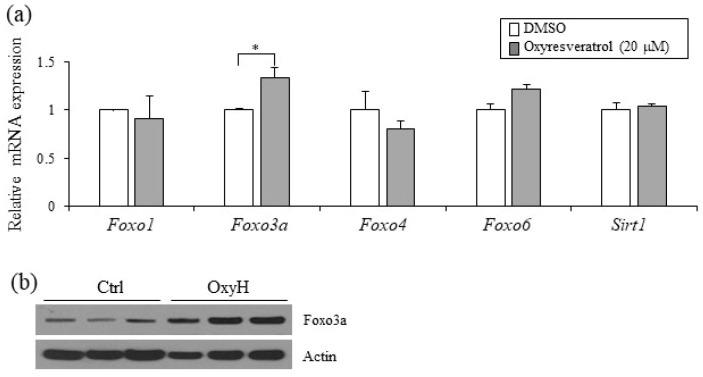
Selective induction of Foxo3a as an oxyresveratrol-regulated gene. (**a**) C3H10T1/2 adipocytes were treated with oxyresveratrol for 12 h and the regulatory effects on Foxo family and *Sirt1* gene expression were analyzed. (**b**) In vivo regulation of Foxo3a protein in HFD-fed obese mice treated with vehicle control (Ctrl) or oxyresveratrol (OxyH) for 8 weeks. Expression of Foxo3a protein in iWAT of control and oxyresveratrol-treated mice was determined by Western blotting. Statistically significant differences were determined using Student’s *t*-test (* *p* < 0.05).

Oxyresveratrol has been shown to regulate the immune system, exhibit neuroprotective effects, extend lifespan, decrease acute liver injury, and prevent obesity [17,18,20]. These oxyresveratrol-mediated biological effects are believed to be mostly related to the activation of a few signaling pathways similar to the mechanism of resveratrol, a structurally analogous bioactive compound. In particular, Sirt1 and Foxo family members stimulated by oxyresveratrol and its analog resveratrol are postulated to exert the effects on Ucp1 and thermogenesis. To delineate the molecular mechanism of oxyresveratrol in adipocytes, we determined the levels of the transcripts Sirt1 and Foxo involved in thermogenesis. Oxyresveratrol treatments induced the expression of Foxo3a but did not induce other Foxo family members (Foxo1, Foxo4, and Foxo6) and Sirt1 levels (Figure 5a). Increased Foxo3a protein expression was also verified in the iWAT from oxyresveratrol-treated mice (Figure 5b and Figure S8), suggesting that increased Foxo3a expression may, at least in part, mediate the actions of oxyresveratrol in adipocytes.

**Figure 6:**
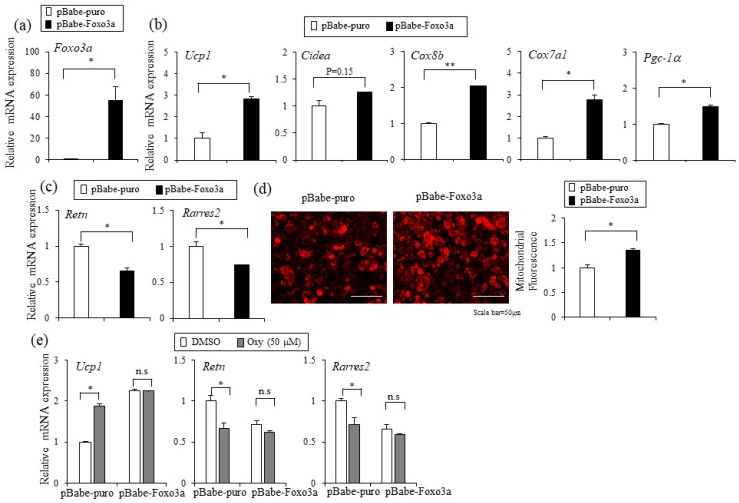
Foxo3a induces expression of Ucp1 and thermogenic genes. (**a**) C3H10T1/2 cells were infected with retrovirus harboring the empty vector control (pBabe-puro) or Foxo3a gene (pBabe-Foxo3a) and large stable pools were selected with puromycin (2 μg/mL). Expression of *Foxo3a* in C3H10T1/2 adipocytes was verified by real-time PCR. (**b**) The effects of Foxo3a stable overexpression on the expression of thermogenic genes including *Ucp1*, *Cidea*, *Cox8b*, *Cox7a1*, and *Pgc-1α* in C3H10T1/2 adipocytes were determined by real-time PCR. (**c**) Effects of stable overexpression of Foxo3a on white adipocyte selective genes (*Retn and Rarres2*) in C3H10T1/2 adipocytes were determined. (**d**) Mitochondrial staining (left) by mitochondria-specific CytoPainter (ab112145) in the control plasmid-expressing (pBabe-puro) and *Foxo3a*-overexpressing (pBabe-Foxo3a) adipocytes. (right) Mitochondrial staining was quantified by NIH Image J software. (**e**) Foxo3a-overexpression abrogates the effects of oxyresveratrol in the regulatory effects on *Ucp1*, *Retn*, and *Rarres2* expression. Data represent means ± s.e.m. and are representative of three independent experiments. Each independent experiment was carried out in triplicate. Statistically significant differences were determined using Student’s *t*-test (* *p* < 0.05; ** *p* < 0.005).

To gain insight into the roles of Foxo3a in adipocytes, we stably overexpressed Foxo3a using the pBabe-retrovirus system. The ectopic expression of Foxo3a in C3H10T1/2 adipocytes enhanced the expression of thermogenic genes Ucp1, Cidea, Cox8b, Cox7a1, and Pgc-1α (Figure 6a,b). Furthermore, Foxo3a also decreased the lipid accumulation and expression of adipocyte markers during adipocyte differentiation, similar to the effects by oxyresveratrol (Figure 6c and Figure S4). This Foxo3a mediated effect is also accompanied with increased mitochondrial mass (Figure 6d). These results suggest that the stable expression of Foxo3a in adipocytes can mimic oxyresveratrol’s actions in inducing thermogenic gene expression, mitochondrial mass, and inhibition of lipid accumulation. To test whether ectopic Foxo3a can synergistically stimulate the oxyresveratrol’s actions, we treated oxyresveratrol in control and Foxo3a stably overexpressing cells. Foxo3a stable overexpression displays the blunted effects of oxyresveratrol on the expression of Ucp1, white selective adipocyte markers (Retn and Rarres2), and pan-adipocyte markers (Pparγ and Fabp4) compared to the levels in the control pBabe-puro expressing cells (Figure 6e and Figure S5). The absence of an additive effect with oxyresveratrol and Foxo3a suggests the common molecular mechanisms. These data suggest the functional importance of Foxo3a in the oxyresveratrol-mediated effects in adipocytes.

**Figure 7:**
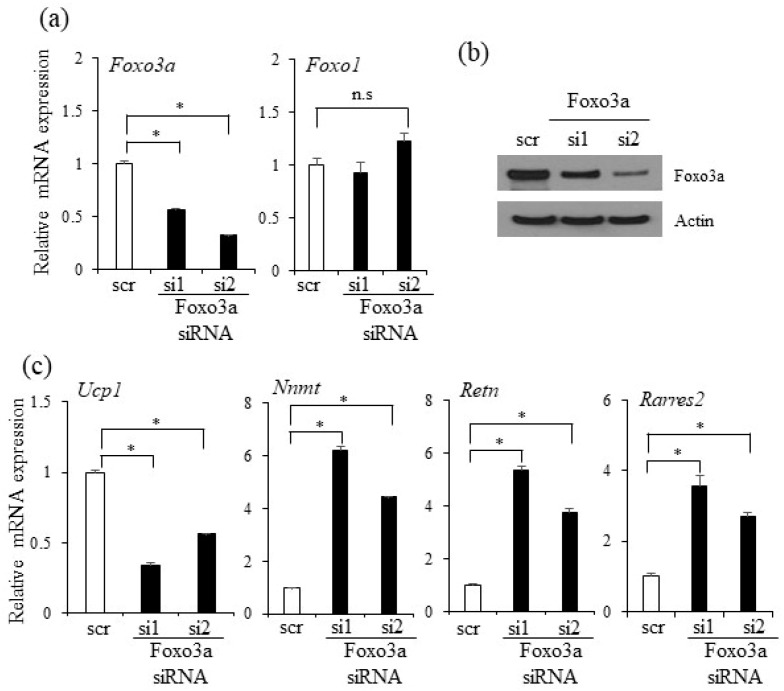
Foxo3a silencing decreases Ucp1 yet increases white adipocyte selective genes in C3H10T1/2 adipocytes. (**a**) Knockdown of Foxo3a by two independent siRNAs (si1 and si2) reduced the expression of *Foxo3a* but not *Foxo1* compared to the scrambled non-specific control (scr) siRNA-transfected C3H10T1/2 cells. (**b**) Two independent Prdm4-specific siRNAs decrease Foxo3a protein expression. (**c**) Knockdown of Foxo3a decreases the expression levels of *Ucp1* yet increases *Nnmt, Retn,* and *Rarres2* mRNA expression compared to the scrambled control (scr) siRNA-transfected cells. Each experiment was carried out in triplicate. Data represent means ± s.e.m. Statistically significant differences were determined using Student’s *t*-test (* *p* < 0.05).

To further investigate the roles of Foxo3a in adipocytes, we silenced the expression of Foxo3a by two siRNAs, effectively reducing Foxo3a, but not the related factors of Foxo1, in C3H10T1/2 adipocytes (Figure 7a,b and Figure S8). The Foxo3a-silenced C3H10T1/2 preadipocytes (si1 and si2) promoted lipid accumulation and induced the expression of the adipocyte markers Pparγ and Fabp4 compared to the scrambled control (scr) siRNA cells (Figure S6). Similar pro-adipogenic effects were also observed in Foxo3a-deficient 3T3-L1 cells (Figure S6). Interestingly, the Foxo3a deficient C3H10T1/2 cells exhibited repressed expression of Ucp1 but increased expression of Nnmt, Retn, and Rarres2 (Figure 7c). Furthermore, Foxo3a knockdown in brown adipocyte T37i cells reduced the expression of thermogenic genes including Ucp1, Cidea, and Cox8b (Figure S7). These results show that Foxo3a can recapitulate the effects of oxyresveratrol for WAT browning and anti-adipogenic effects. These results suggest that Foxo3a has pathophysiological functions in obesity and metabolic diseases, and further suggest Foxo3a as a regulator of oxyresveratrol-mediated thermogenic programming.

## 3. Discussion

Oxyresveratrol is a natural polyphenol isolated from Artocarpus lackocha Roxb and Morus alba L. (mulberry) [21]. It exhibits anti-inflammatory, anti-viral, anti-oxidative, and neuroprotective activities [15,22,23,24]. In addition, recent studies showed that an HFD supplemented with oxyresveratrol lowered body-weight gain without affecting the food intake, suggesting that oxyresveratrol may induce energy expenditure in HFD-fed obese mice. Further studies showed that oxyresveratrol-induced key metabolic genes in mouse tissues. It induces the expression of IRS-1 and Glut4 in adipose tissue and muscles, G-6-Pase, SREBP-1, FAS, C/EBPα, and AMPKα2 in the liver [18]. However, the primary cause of oxyresveratrol in alleviating obesity and glucose metabolism is not currently known. In this study, we showed that oxyresveratrol increased O_2_ consumption and CO_2_ production even before body-weight differences are evident. The increased energy expenditure is not associated with affecting energy intake and physical activities, indicating that the higher energy expenditure by oxyresveratrol can lead to the prevention of obesity and its associated metabolic diseases. Taken together, enhanced energy expenditure in adipose tissue may account for the effects of oxyresveratrol in preventing obesity and its associated metabolic dysregulation.

In this study, we showed that oxyresveratrol can exert anti-obese effects probably through the induction of thermogenesis and energy expenditure. The remaining question is how oxyresveratrol regulates the expression of thermogenic genes. Since Ucp1 is a known major thermogenic player in mitochondria, Ucp1 may function as a mediator of oxyresveratrol. Indeed, we observed Ucp1 induction in the adipocytes and WAT of oxyresveratrol-treated mice. Thermogenic adipocytes, including brown and beige adipocytes, ameliorate obesity and metabolic diseases through increases of Ucp1 mediated thermogenesis and energy expenditure [6,25]. The regulation of Ucp1 can be achieved through the cooperation of various stimuli and mediators in metabolic and pathophysiological contexts. Cold exposure followed by β-adrenergic-induced thermogenesis causes the activation of WAT and BAT, Ucp1 stimulation, and the induction of thermogenic programming [7,10,11]. Regulation of signaling pathways including those of AMPKα, PKA, Sirt1, and JAK-STAT, or the transcriptional activation of Pgc1α, Ppar, TLE3, EBF, Prdm4, Prdm16, and Foxos has been shown to stimulate thermogenesis [26,27,28,29,30,31,32,33,34,35,36,37]. In this study, we showed that oxyresveratrol selectively induced Foxo3a, but did not induce other Foxo family members. Although it is not known which biological inputs affect Foxo3a expression in adipocytes, our data imply that oxyresveratrol-mediated Foxo3a signaling can contribute to Ucp1 induction and stimulation of energy expenditure. The identification of the direct regulatory effects of oxyresveratrol in thermogenic genes may resolve the thermogenic mechanism and thus is an intriguing question.

Resveratrol is a structurally similar to oxyresveratrol and a relatively well-known phytochemical with four hydroxyl groups on its aromatic ring. It has been shown to prevent body-weight gain and glucose metabolism [38]. Resveratrol enhances brown adipogenic markers in stromal vascular cells possibly through Sirt1, AMPKα1 or Foxo activation [26,39]. It is thus possible that resveratrol can also induce the browning of WAT to increase energy expenditure, similar to the molecular mechanisms used by oxyresveratrol. However, it has been shown that, compared to resveratrol, oxyresveratrol is a superior antioxidant and has free radical scavengers, but has less cytotoxicity [40]. Therefore, slight modifications on the bioactive compounds can produce somewhat different biological outcomes. Resveratrol was able to induce Foxo3a expression. Therefore, it is possible that these two bioactive compounds share molecular mechanisms, but with slightly different capability in WAT browning and anti-obese actions. However, it is still plausible that resveratrol and oxyresveratrol may use their unique and alternative molecular mechanisms besides Foxo3a for the stimulation of brown adipocytes and energy expenditure, and this should be further explored.

While oxyresveratrol did not affect energy intake, physical activities, fecal weight, and lipid content, it increased metabolic rates and rectal temperature, suggesting that oxyresveratrol prevents obesity and associated diseases through, at least in part, the stimulation of browning adipocytes. However, the current study did not exclude the possibility of oxyresveratrol in other metabolic tissues including liver and muscles. Indeed, previous studies have shown that oxyresveratrol decreases hepatic oxidative stress and hepatosteatosis [17]. In addition, the liver has been shown to participate in the thermogenic effects during cold exposure in mice [41]. These observations suggest the possible contribution of other tissues for the beneficial effects of oxyresveratrol in energy expenditure. These observations warrant further study to determine the direct effects of oxyresveratrol in other metabolic tissues.

In this study, we treated obese mice with an intraperitoneal injection of oxyresveratrol and showed its effects on energy expenditure and obesity. Previous studies have shown that the oral delivery of oxyresveratrol prevents obesity without affecting food intake [18]. Therefore, it is likely that the oral delivery of oxyresveratrol might have increased Ucp1 expression and enhanced energy expenditure, similar to the current finding. Based on these, oxyresveratrol in obesity related metabolic diseases such as fatty liver diseases and diabetes, and especially in its human application, would be another avenue to broaden its uses in the future.

## 4. Materials and Methods

### 4.1. Cell Culture and Adipocyte Differentiation

C3H10T1/2 and 3T3-L1 cells were purchased from the American Type Culture Collection (Rockville, MD, USA) and were maintained as previously described [42]. 3T3-L1 cells were induced into adipocytes in Dulbecco’s modified Eagle’s medium (DMEM) (Hyclone, Logan, UT, USA) media supplemented with 10% FBS, 1 μM dexamethasone (Sigma, St. Louis, MO, USA), 0.5 mM isobutyl-1-methylxanthine (Sigma, St. Louis, MO, USA) and 5 μg/mL insulin (Sigma). After 48 h, the differentiating cells were refreshed with media containing DMEM, 10% FBS and 5 μg/mL insulin. C3H10T1/2 cells were further supplemented with 20 nM GW1929 (Sigma) to induce adipocyte differentiation. T37i cell lines, a brown preadipocytes, kindly provided by M. Lombes (Paris-Sud University, Le kremlin Bicêtre, France) were maintained DMEM/F-12, 10% CS, and 1% penicillin/streptomycin. Confluent cells were incubated in DMEM/F-12, 10% CS, 5-μg/ml insulin and 2.5 nM T3, to induce brown adipocytes. After adipocyte differentiation for 6–8 days, cells were fixed with 4% paraformaldehyde in phosphate-buffered saline (PBS) and stained with 0.5% Oil Red O (Sigma).

### 4.2. Cell Viability Assays

Cell viability was determined using the 3-(4,5-dimethylthiazol-2-yl)-2,5-diphenyltetrazolium -bromide (MTT, Sigma). 3T3-L1 cells were seeded at 1.5 × 104 cells per well in 96-well plates and treated with various doses of oxyresveratrol (Tokyo Chemical Industry, Tokyo, Japan) in triplicate. After 48 and 96 h, MTT (5 mg/mL in PBS) was added into media and were incubated at 37 °C for an additional 4 h. The formazan crystals were dissolved in 200 μL DMSO, and the absorbance was measured at 520 nm using a microplate reader (BioTek, Winooski, VT, USA).

### 4.3. Expression Analysis

Total RNA was isolated from the cells or tissues using TRIzol reagent (Invitrogen, Carlsbad, CA, USA). First-strand cDNA (cDNA) was synthesized using ReverTra Ace^®^ qPCR RT Master Mix (TOYOBO, Osaka, Japan) with random primers. The cDNA with THUNDERBRID® SYBR^®^ qPCR Mix (TOYOBO) and primers was amplified using the Applied Biosystems QuantStudio 3 Real-Time PCR (Foster city, CA, USA). Expression was normalized to 36b4. Gene specific primer sets were described previously [36,43]. Total RNA from adipose tissues was isolated using RNeasy Lipid Tissue Mini Kit (Qiagen, Hilden, Germany).

For protein expression analysis, adipocytes and adipose tissues were harvested and lysed in radioimmunoprecipitation assay (RIPA) buffer (1% NP-40, 50 mM Tris-HCl, pH 7.4, 150 mM NaCl, and 10 mM NaF) containing a protease inhibitor cocktail (Roche Diagnostics, Risch-Rotkreuz, Switzerland). The proteins were separated on SDS-PAGE and transferred to nitrocellulose membranes (Bio-Rad Laboratories, Hercules, CA, USA). The membranes were blocked with 5% skim milk and treated with anti-Ucp1 (ab10983, Abcam, Cambridge, UK), anti-PGC1alpha (ab54481, Abcam), anti-PRDM16 (ab106410, Abcam), anti-Foxo3a (Cell Signaling Technology, Danvers, MA, USA), or anti-Actin (sc47778, Santa Cruz Biotech, Dallas, TX, USA) antibodies followed by detection with Enhanced Chemiluminescence Western Blotting Detection Reagent (Amersham Biosciences, Little Chalfont, UK).

### 4.4. Knockdown and Overexpression Studies

The scramble control, forkhead box o3a (FoxO3a)-specific small interfering RNA (siRNA) were synthesized by Genolution Pharmaceuticals, Inc. (Seoul, Korea). Two independent siRNA (si1 and si2) sequences are 5′-GACAAACGGCUCACUUUGUUU-3′ and 5′-GAACAGAACUCUAUAACAU UU-3′. Cells were seeded at a density of 1×105 cells per well in a 6-well plate and transfected with 30 nmol of scrambled RNA or Foxo3a-specific siRNA using RNAiMAX (Invitrogen) as previously described [29,36]. After 8 h, the cells were refreshed with the fresh media and further induced to adipocyte differentiation.

HA-FoxO3a from Addgene (Cambridge, MA, USA) was cloned into pBabe-puro vector. For stable overexpression, 293T cells were co-transfected with retroviral packaging vectors (Gag-Pol, VSV-G) and pBabe-puro or pBabe-puro-FoxO3a. At 48 h post-transfection, viral supernatants with polybrene (8 ng/mL, Sigma) were harvested and filtrated. Cells were infected with the viral supernatants and stable cells were selected with treatments of puromycin (4 μg/mL) for 2 weeks.

### 4.5. Animal Studies

Male C57BL/6N mice (7 weeks old) were obtained from Japan SLC, Inc (Hamamatsu, Shizuoka, Japan). The mice were individually housed in a temperature-controlled room and 12 h light/dark cycle. After 1 week of adaptation, the mice were randomly divided into four groups: normal diet (ND, 10% fat w/w), HFD, 60% fat w/w) injected with vehicle (3% ethanol in PBS) control (Ctrl), and HFD treated with two doses of oxyresveratrol with 3% of ethanol in PBS (OxyL, 7.5 mg/kg/day or OxyH, 15 mg/kg/day). Body weight and food intake were measured twice per week. For glucose tolerance test, the mice treated for 8 weeks were fasted for 16 h, and blood glucose was determined from tail-vein blood at 0, 15, 30, 60, 90, and 120 min after i.p. (intraperitoneal injection) glucose injection (2 g/kg). The blood glucose level was measured by Blood Glucose Monitor Nocoding 1 Plus (GM01BAA. DAEIL PHARM Co., Ltd., Sungnam, Korea). For insulin tolerance test, the mice were injected i.p. with insulin (Humulin R, Eli Lilly, Indianapolis, IN, USA) (0.35 U/kg). All animal studies were carried out in accordance with the guidelines of the Animal Research Committee (SKKUIACUC-20150037) of Sungkyunkwan University.

### 4.6. Metabolic Studies

For mitochondrial staining, CytoPainter (ab112145; Abcam) was used and added to live cells and incubated for 1 h before fixation. The CytoPainter stained cells were observed by fluorescence microscopy and quantified as previously described [36].

Whole-body energy metabolism was measured using Oxylet systems (Panlab, Barcelona, Spain). When body weights were not different (treated with oxyresveratrol for 3 weeks), mice were placed in metabolic cages and acclimated for 20–24 h and measured for oxygen consumption, carbon dioxide release, and energy expenditure.

### 4.7. Temperature Measurements

Rectal temperature was measured from mice treated with control or oxyresveratrol for 8 weeks using a temperature thermometer (Testo 925 Digital Thermometer, Order-Nr. 0560 9250, Testo, Germany) at 10 AM.

### 4.8. Collection of Feces and Extraction of Lipid

Mice were individually housed for 24 h and feces were collected, dried, and weighed. The feces were grinded and total lipids were extracted using chloroform: methanol (2:1) and weighed as previously described [44].

### 4.9. Statistical Analysis

Data are presented as mean ± s.e.m. Comparisons between the control and experiment groups were analyzed using a two-tailed unpaired Student’s *t*-tests. Statistical significance was defined as *p* < 0.05.

## Supplementary Materials

Supplementary materials can be found at http://www.mdpi.com/1422-0067/20/1/26/s1.

## Abbreviations

PPARγ	Peroxisome proliferator-activated receptor γ
Fabp4	Fatty acid binding protein 4
siRNA	Small interfering RNA
MSC	Mesenchymal stem cells
DMI	Dexamethasone, IBMX, and Insulin
AMPK	AMP- activated protein kinase
Ucp1	Uncoupling protein1
WAT	White adipose tissue
BAT	Brown adipose tissue
Myf5	Myogenic factor (Myf5)
Pax7	Paired box 7
En1	Engrailed 1
PDGF- β	Platelet-derived growth factor–β
SMA	Smooth muscle
Myh11	Myosin heavy chain 11
Prdm16	PR domain containing protein 16
Pgc-1α	Peroxisome proliferator-activated receptor gamma coactivator-1α
TRPV1	Transient receptor potential vanilloid 1
HFD	High-fat diet
Foxo3a	Forkhead box o3a
Retn	Resistin
Nnmt	Nicotinamide N-Methyltransferase
Rarres2	Retinoic acid receptor responder protein 2
Cidea	Cell death-inducing DFFA-like effector a
Elovl3	Elongation of very long chain fatty acids protein 3
Sirt1	Sirtuin 1
Cox7a1	Cytochrome c oxidase polypeptide 7a1
Cox8b	Cytochrome c oxidase subunit 8b
