# Supplementary material for: Oxyresveratrol Increases Energy Expenditure through Foxo3a-Mediated Ucp1 Induction in High-Fat-Diet-Induced Obese Mice
Source: Int J Mol Sci. 2018 Dec 21;20(1):26. doi: 10.3390/ijms20010026 (PMC6337118; doi:10.3390/ijms20010026)
Supplement: Supplementary file 1 [file ijms-20-00026-s001.pdf]

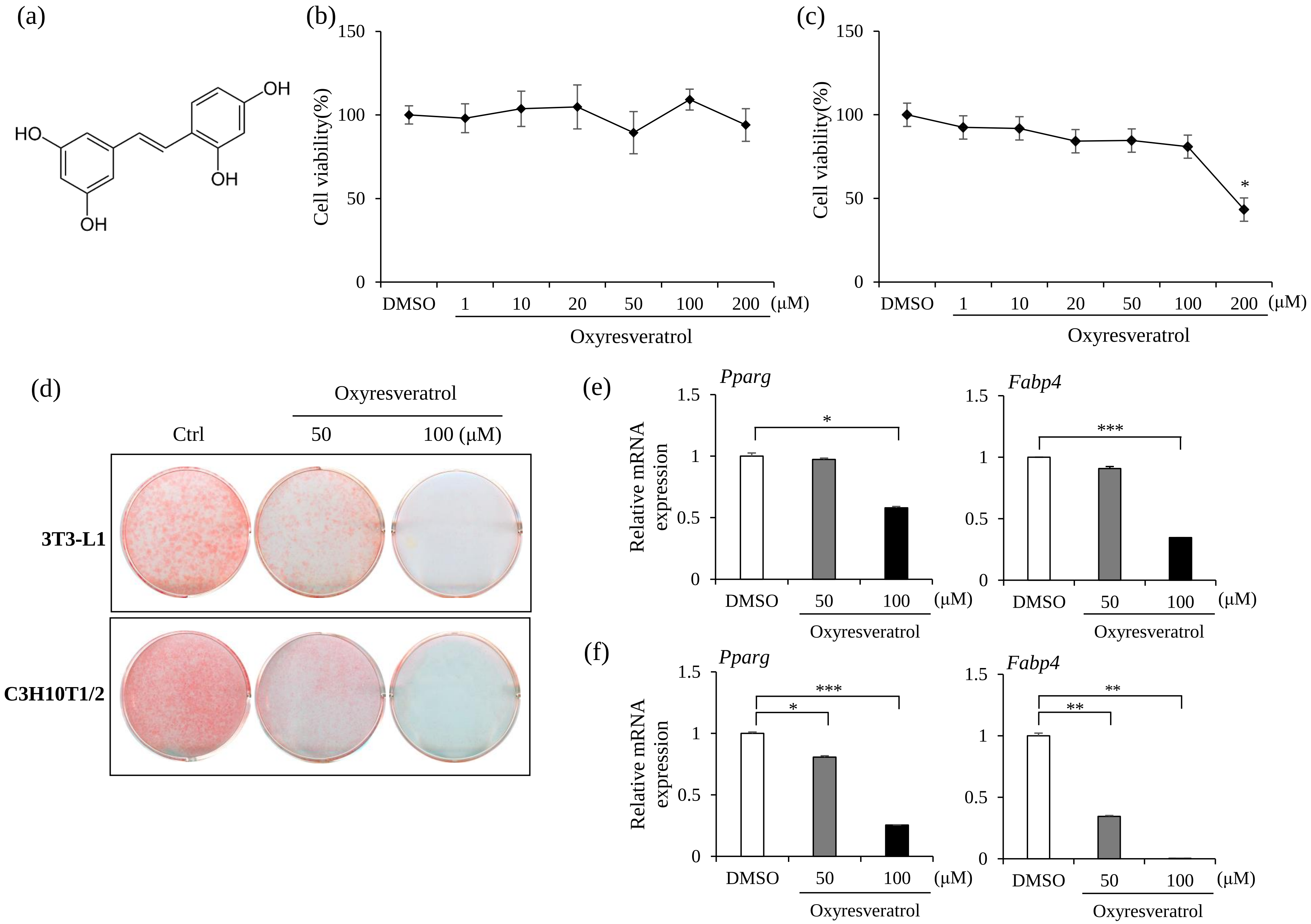
(a)
HO
OH
OH
OH
(b)
Cell viability(%)
150
100
50
0
DMSO
1
10
20
50
100
200
(μM)
Oxyresveratrol
(c)
Cell viability(%)
150
100
50
0
*
DMSO
1
10
20
50
100
200
(μM)
Oxyresveratrol
(d)
Oxyresveratrol
Ctrl
50
100 (μM)
3T3-L1
C3H10T1/2
(e)
Pparg
Relative mRNA expression
1.5
1
0.5
0
*
DMSO
50
100
(μM)
Oxyresveratrol
Fabp4
1.5
1
0.5
0
***
DMSO
50
100
(μM)
Oxyresveratrol
(f)
Pparg
Relative mRNA expression
1.5
1
0.5
0
***
*
DMSO
50
100
(μM)
Oxyresveratrol
Fabp4
1.5
1
0.5
0
**
**
DMSO
50
100
(μM)
Oxyresveratrol

Supplementary Figure S1. Oxyresveratrol decreases lipid accumulation and adipocyte differentiation. (**a**) Chemical structure of Oxyresveratrol. (**b**) 3T3-L1 cells were treated with dimethylsulfoxide (DMSO) or oxyresveratrol at the indicated doses for 48 hours and cell viability was determined by MTT assays. (**c**) 3T3-L1 were treated with oxyresveratrol for 96 hours and MTT assays were performed. (**d**) 3T3-L1 preadipocytes or C3H10T1/2 cells were treated with oxyresveratrol at the indicated doses for 6 days and lipid accumulation was determined by Oil Red O staining. Expression levels of *Pparγ* and *Fabp4* mRNA in 3T3-L1 (**e**) or C3H10T1/2 cells (**f**) were quantified by real time PCR. Data shown represent means ± s.e.m. Statistical significance was determined relative to a control using Student's t-test (* $P<0.05$; ** $P<0.005$; *** $P<0.0005$).

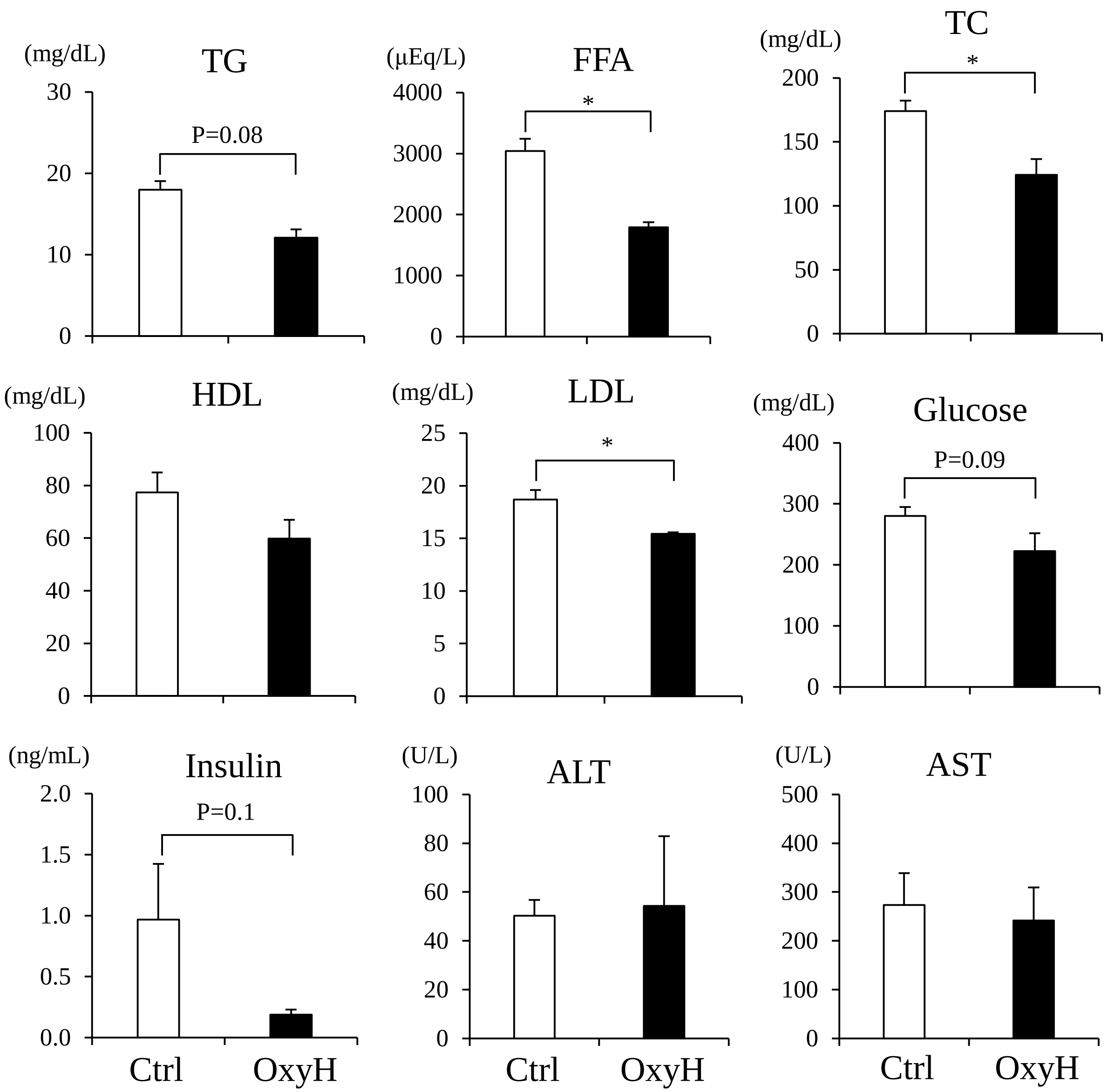

Supplementary Figure S2. Effects of oxyresveratrol in HFD-fed obese mice on serum lipid profiles. Plasma concentrations of triglycerides (TG), free fatty acids (FFA), total cholesterol (TC), high density lipoprotein (HDL), low density lipoprotein (LDL), glucose, insulin Alanine aminotransferase (ALT), and aspartate transaminase (AST) levels in mice fed HFD and administered vehicle control (Ctrl) or a daily dose of oxyresveratrol at 15 mg/kg (Oxy H). Ctrl (N = 6), Oxy H (n=6). Data represent means ± s.e.m. Statistically significant differences in the control and oxyresveratrol-treated mice were determined using Student's *t*-test (* $P < 0.05$; ** $P<0.005$).

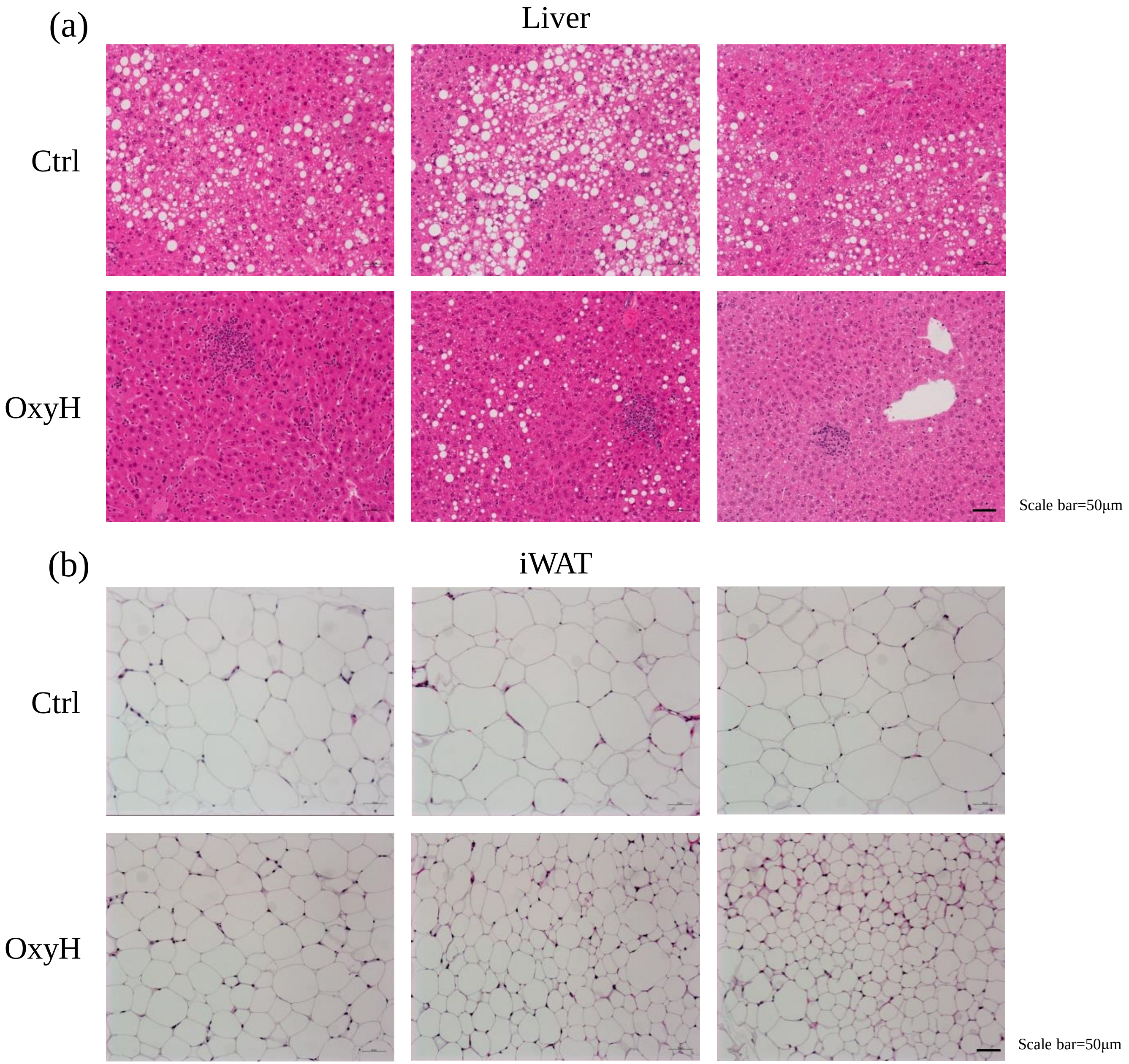

Supplementary Figure S3. Effects of oxyresveratrol on histological changes in HFD fed obese mice. Three representative hematoxylin and eosin (H&E) staining for sections of liver (**a**) and inguinal fat (iWAT) (**b**) from HFD-fed control (Ctrl) or oxyresveratrol (15mg/kg, Oxy H) treated mice. Scale bar, 50 μm.

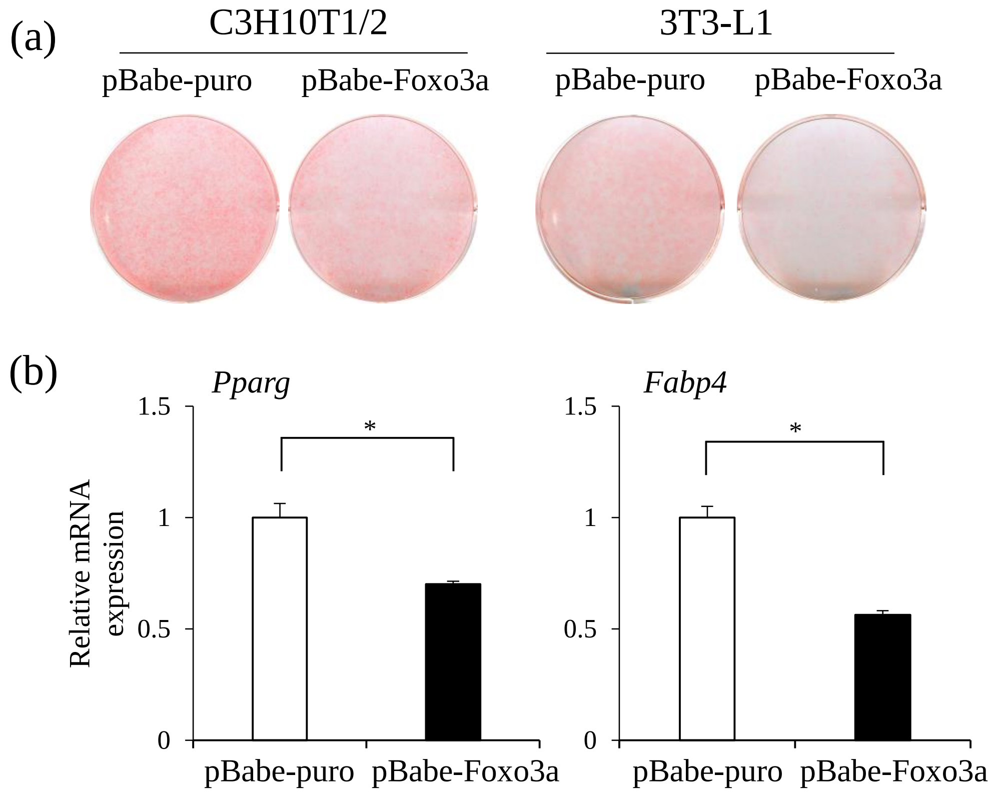

Supplementary Figure S4. Ectopic expression of Foxo3a decreases lipid accumulation and expression of adipocyte markers. (**a**) C3H10T1/2 cells (left) and 3T3-L1 (right) were infected with retrovirus harboring the vector control or Foxo3a gene and large stable pools were selected with puromycin (2 µg/ml). Stable cells expressing control empty vector (pBabe-puro) or Foxo3a (pBabe-Foxo3a) cells were differentiated into adipocytes for 6 days and lipid accumulation was assessed by Oil red O staining. Data are representative of three independent experiments. (**b**) Expression of *Pparγ* and *Fabp4* was measured in pBabe-puro and pBabe-Foxo3a C3H10T1/2 stable cells by real time PCR. Each experiment was carried out in triplicate. Data represent means ± s.e.m. Statistically significant differences were determined using Student's *t*-test (* $P<0.05$).

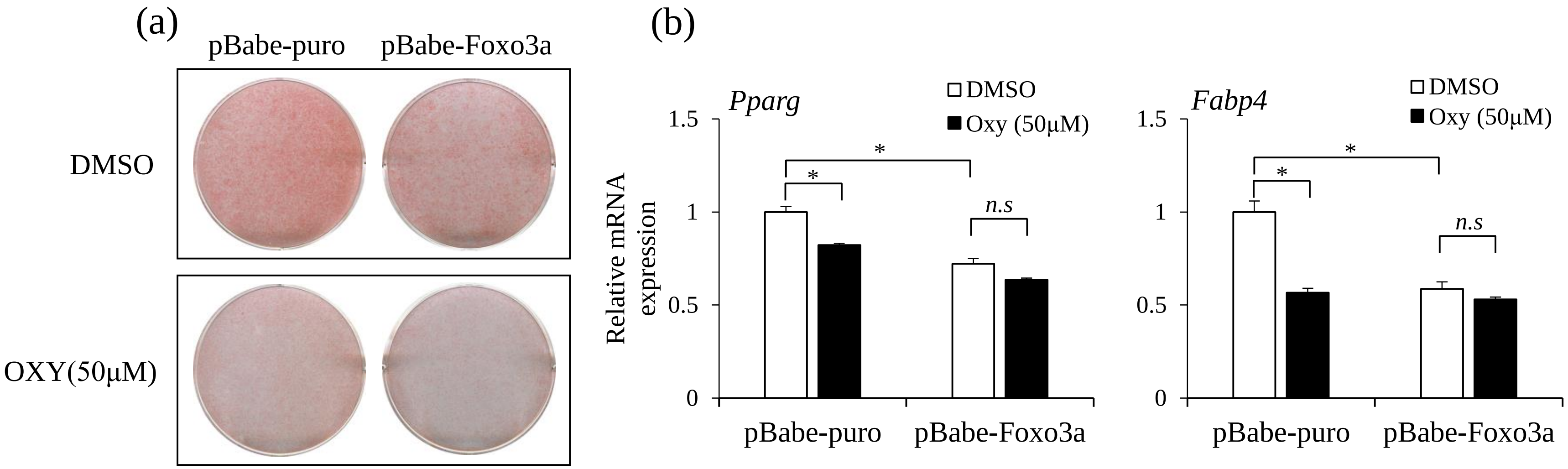

Supplementary Figure S5. Stable overexpression of Foxo3a weakens the inhibitory effects of oxyresveratrol on lipid accumulation and adipocyte marker expression. (**a**) Stable cells expressing control empty vector (pBabe-puro) or Foxo3a (pBabe-Foxo3a) cells were differentiated into adipocytes and treated with oxyresveratrol (50 μM) for 6 days followed by Oil red O staining. Data are representative of three independent experiments. (**b**) Stable cells expressing control empty vector (pBabe-puro) or Foxo3a (pBabe-Foxo3a) cells were differentiated and treated with oxyresveratrol for 6 days. Effects of oxyresveratrol in control empty vector (pBabe-puro) or pBabe-Foxo3a cells on mRNA expression of *Ppar*γ and *Fabp4* were determined by real time PCR. Data are representative of two independent experiments. Data represent means ± s.e.m. Statistically significant differences were determined using Student's *t*-test (* $P < 0.05$; NS, not significant).

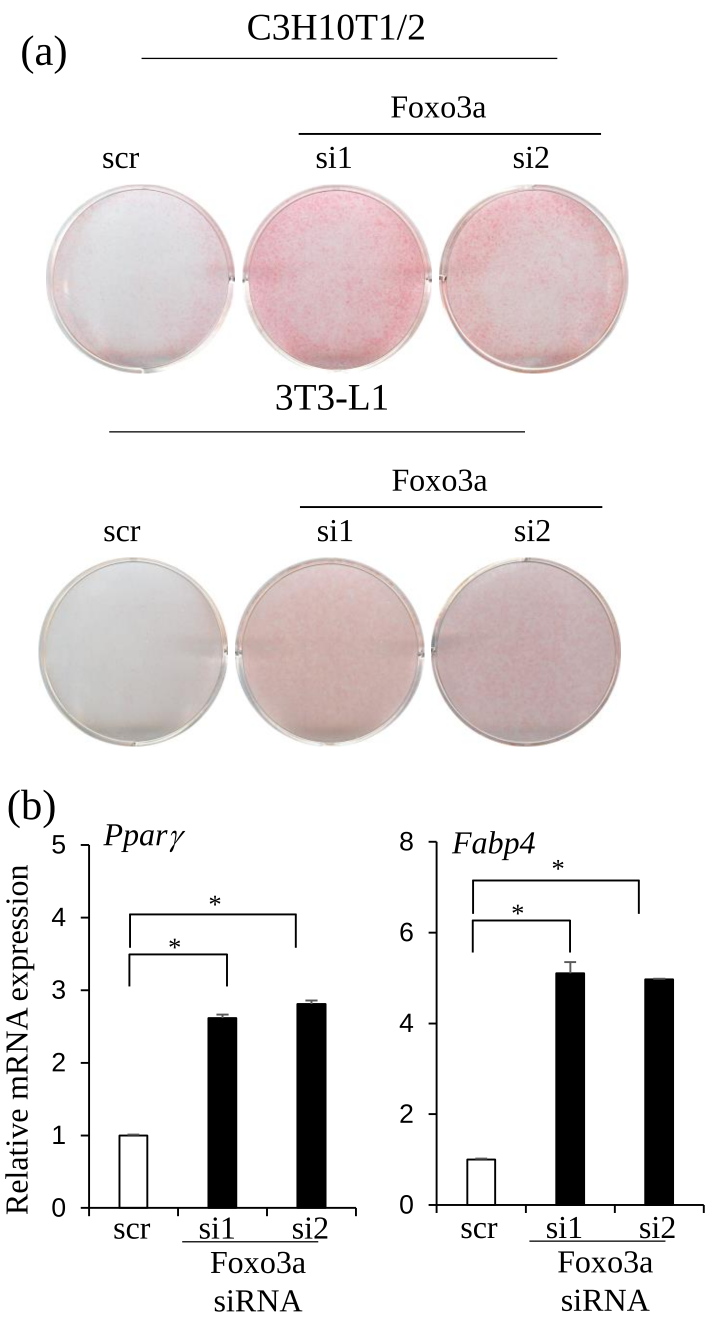

(a)
C3H10T1/2
Foxo3a
scr
si1
si2
3T3-L1
Foxo3a
scr
si1
si2
(b)
Pparγ
Fabp4
Relative mRNA expression
5
4
3
2
1
0
8
6
4
2
0
*
*
*
*
scr
si1
si2
Foxo3a siRNA
scr
si1
si2
Foxo3a siRNA

Supplementary Figure S6. Foxo3a silencing promotes lipid accumulation and expression of adipocyte markers. (**a**) Transient transfection of scrambled non-specific control (scr) or two independent Foxo3a-targeting siRNAs (si1 and si2)-transfected or C3H10T1/2 (top) or 3T3-L1 cells (bottom) were differentiated into adipocytes for 6 days and lipid accumulation was assessed by Oil red O staining. Data are representative of three independent experiments. (**b**) Knockdown of Foxo3a by siRNAs in C3H10T1/2 cells increases expression of *Ppar*γ and *Fabp4*. Each experiment was carried out in triplicate. Data represent means ± s.e.m. Statistically significant differences were determined using Student's *t*-test (* $P<0.05$).

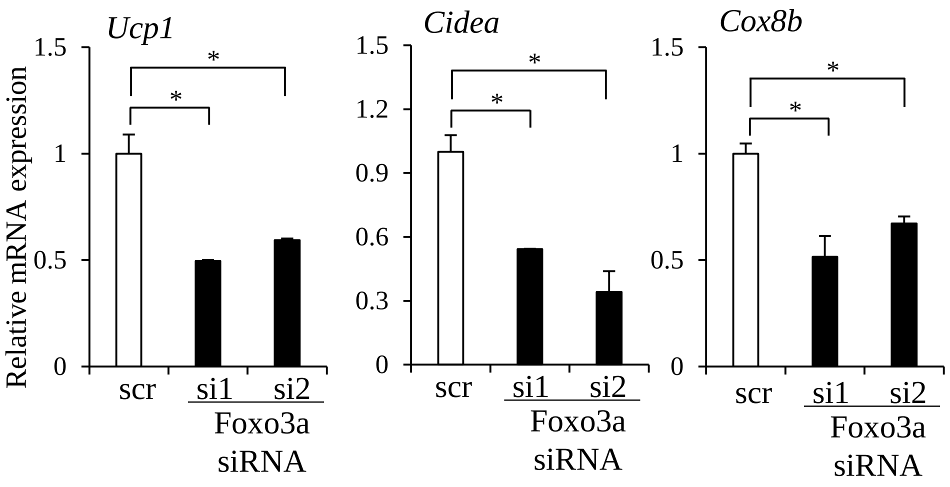

Supplementary Figure S7. Foxo3a silencing promotes expression of white adipocyte selective genes in T37i brown adipocytes. Transient transfection of scrambled non-specific control (scr) or two independent Foxo3a-targeting siRNAs (si1 and si2)-transfected prebrown T37i cells were differentiated into brown adipocytes for 6 days and the expressions of brown adipocyte selective markers, *Ucp1*, *Cidea*, and *Cox8b* were measured by real time PCR. Each experiment was carried out in triplicate. Data represent means ± s.e.m. Statistically significant differences were determined using Student's *t*-test (* $P<0.05$).

Original blots

Figure 3a

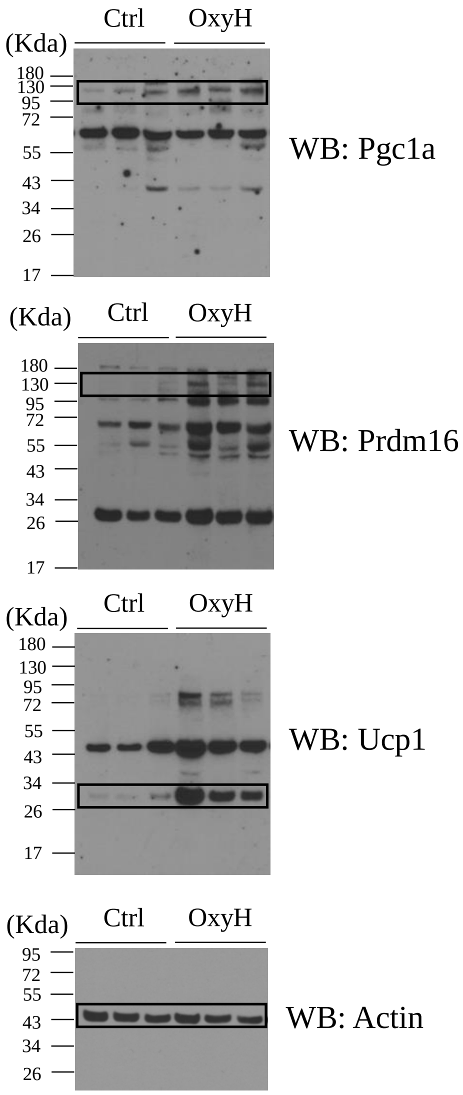

Figure 5b

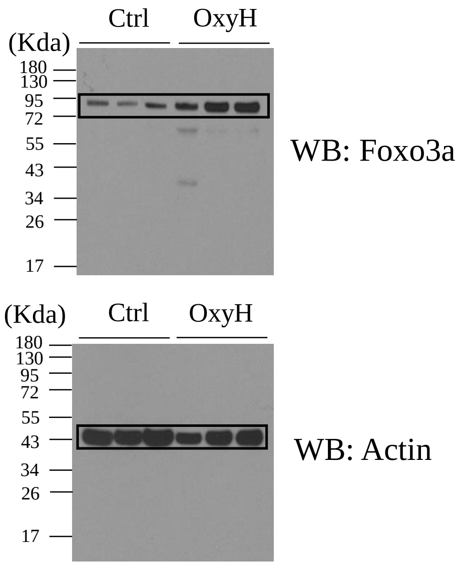

Figure 7b

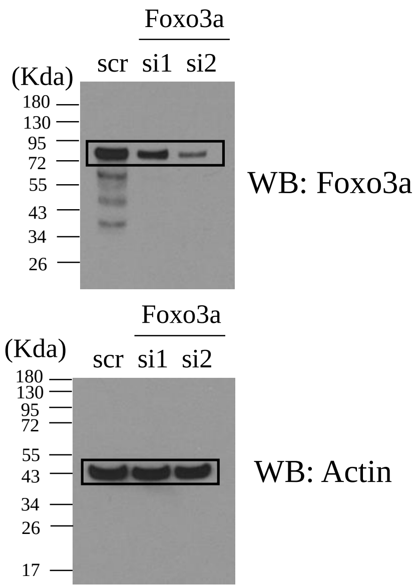

Supplementary Figure S8. Uncropped versions of immunoblots in the figure 3a, 5b, and 7b
